# Participation in Daily Activities of Children with Atopic Diseases and Its Relation to Their Sensory Modulation Difficulties

**DOI:** 10.3390/children11111300

**Published:** 2024-10-27

**Authors:** Batya Engel-Yeger, Aharon Kessel

**Affiliations:** 1 Occupational Therapy Department, Faculty of Social Welfare and Health Sciences, University of Haifa, Haifa 3498838, Israel; bengel@univ.haifa.ac.il; 2 Division of Allergy and Clinical Immunology, Bnai Zion Medical Center, Rappaport Faculty of Medicine, Technion, Israel Institute of Technology, Haifa 3104802, Israel

**Keywords:** atopic dermatitis, asthma, rhinitis, children, sensory modulation, participation, daily activities

## Abstract

(1) Background: Participation in daily activities is critical for a child’s health, development and wellbeing and is considered a main outcome measure of intervention efficiency. Atopic diseases affect children’s daily life and routines but the knowledge about impacts on participation is limited. (2) Objectives: a. to profile the preference to participate in daily activities of children with atopic diseases as compared to healthy controls; b. to profile sensory modulation difficulties (SMD) in each atopic group; c. to examine whether SMD correlate with activity preference. (3) Methods: This cross-section and correlative study included 253 children aged 4–11: 37 with Asthma, 37 with atopic dermatitis (AD), and 31 with Rhinitis. The controls were 148 healthy children. All children completed the Preference for Activities of Children (PAC) while their parents completed a socio-demographic questionnaire and the Short Sensory Profile (SSP). (4) Results: Children with atopic diseases showed significantly lower preference to participate in activities than healthy controls and preferred quiet activities that can be performed alone. Within the atopic groups, children with AD, and specifically girls, had the highest activity preference while boys with Asthma had the lowest preference. SMD was significantly more prevalent in children with atopic diseases and correlated with their reduced preference to participate in activities. (5) Conclusions: Atopic diseases may restrict children’s participation. Clinicians should evaluate participation of children with atopic diseases and examine whether comorbidities as SMD affect participation. Understanding the implications on children’s daily life may improve intervention efficiency and elevate development and wellbeing.

## 1. Introduction

Based on the International Classification of Functioning, Disability and Health (ICF) model of the World Health Organization [1] it is crucial for health providers to understand the disease implications on the person’s participation in daily activities and on quality of life. Therefore, participation and quality of life are considered as the main outcome measures of intervention. Participation is the involvement in life situations resulting from the interaction of individuals with their social and physical environments. One of the major determinants of participation in leisure and recreation activities is individual preferences or interests [2]. Participation leads to life satisfaction, to a sense of competence and it contributes to the child’s psychological, emotional, and skill development. Therefore, the concept of participation is becoming increasingly important in pediatrics intervention programs [3].

The present study focused on children with atopic diseases: Atopic Dermatitis (AD), Allergic Rhinitis (AR) and Asthma. Atopic diseases, encompassing atopic dermatitis, asthma, and allergic rhinitis, exhibit overlapping symptoms attributable to their shared allergic and inflammatory pathophysiology. Atopic dermatitis is characterized by pruritus, xerosis, and erythematous rashes, which can vary in distribution according to age. Chronic scratching often leads to lichenification. Patients with asthma typically present with wheezing, dyspnea, chest tightness, and coughing, which may be exacerbated during nocturnal hours or early morning. Allergic rhinitis is defined by hallmark symptoms such as sneezing, nasal congestion, rhinorrhea, and pruritus affecting the eyes, nose, or throat. The symptomatic overlap among these conditions reflects the interconnected underlying mechanisms of atopy and inflammation [4,5,6].

Atopic diseases are frequently related to poor adherence to treatment and are known to limit participation in life activities [7]. Hence, their related functional disabilities in daily life should be explored. The complexity and chronicity of atopic diseases in children may restrict their ability to participate in daily activities [8,9]. For example, AD, asthma and allergic rhinitis may decrease children’s participation in physical and social activities [10,11,12]. Moreover, comorbidities of atopic diseases may contribute to the limited participation. For example, sleeping problems may cause children with AD or children with asthma to be more engaged in sedentary activities than in physical activities. This, in turn, may affect their physical status [13,14].

In our studies on participation of children with atopic diseases, similar participation patterns were found in children with AD and children with AR [8,12]. Their restricted participation correlated with an interesting phenotype that was found to characterize children with atopic diseases, named *Sensory Modulation Difficulties* (SMD) [15,16,17]. SMD result in hyper or hypo sensitivity to daily sensory stimuli due to inefficient processing of sensory input in the central nervous system. SMD is associated with hyperarousability as manifested in sleep problems, emotional dysregulation [18], hyperactivity [19], that are also known as be common comorbidities in atopic diseases [20]. We found that SMD is prevalent in about one third of children with various atopic diseases, with no difference between genders. SMD may result from genetic factors [21,22] and significantly impair participation in daily activities [23]. Children with AD and SMD were found to prefer to participate in quiet recreational activities performed alone, such as: doing puzzles, crafts, drawing, playing computer or video games [8]. Nonetheless, studies about participation of children with atopic diseases and specifically about the relations between the disease comorbidities as SMD and participation, are relatively low. There is a lack of knowledge that emphasizes commonalities between atopic groups or unique characteristics of each atopic group in terms of participation and its relation to SMD.

The present study aimed: a. to profile the preference to participate in daily activities of children with atopic diseases as compared to healthy controls; b. to profile sensory modulation difficulties (SMD) in each atopic group; c. to examine whether SMD correlate with activity preference.

These aims might provide answers to the following research questions: Do atopic diseases restrict children’s participation? In which domains? Is there a relationship between participation and other comorbidities of atopic conditions, as SMD?

Hypotheses: (1) Preference to participate in activities would be significantly lower in children with atopic diseases as compared to healthy controls (2) No significant differences would be found between the three atopic groups in their preference to participate in activities. Gender effect would be found (3) Among children with atopic diseases SMD would significantly correlate with preference to participate in daily activities.

## 2. Materials and Methods

### 2.1. Participants

G-Power analysis [23], with effect size of 0.25, an alpha error probability of 0.05 and a power value of 0.8, indicated that 180 participants should be included in the study (45 in each group). This cross-section and correlative study included 253 children aged 4–11: 37 with Asthma, 37 with atopic dermatitis (AD), and 31 with Rhinitis. The controls were 148 healthy children (Table 1). A child was defined as having AD, AR or Asthma if the parents informed that the diagnosis was confirmed by a dermatologist, allergist or pediatric pulmonologist. We did not differentiate patients by disease severity.

When examining socio-demographic parameters, children with AD were significantly younger than the controls (*p* < 0.038) and then children with Asthma (*p* < 0.0001). Children with AR were significantly older than children with Asthma (*p* < 0.0001) and then healthy controls (*p* < 0.0001). Therefore, age was held as a covariate. In the AD group, the number of girls was higher than the boys, while in the other groups the number of boys was higher than the number of girls (*p* = 0.035). We referred to gender effect in the further 2 way MANOVA test. No significant differences were found between groups in fathers’ and mothers’ education level.

All children in the study groups were recruited from the Pediatric Dermatology Clinic at [BLINDED]. The controls were recruited through an advertisement posted in neighborhoods in the same geographic area. All participants learned in the regular education system. Children who were on permanent medication, suffered from Attention Deficit Hyperactivity Disorder, had positive neurological findings, chronic disease that affected their daily function, or developmental disorders, were excluded.

### 2.2. Measures

A sociodemographic questionnaire—to gather relevant information about the child and the family.

The “Preference for Activities of Children” (PAC) [24]—evaluates which activities children/youth prefer to perform outside mandated school hours. Each activity is presented by a picture on a card. There are scores for five activity types: recreational (doing puzzles; collecting things; playing with pets, etc.), active physical (team sport; martial art; participating in school clubs, etc.), social (going to a party; visiting; hanging out, etc.), skill-based (taking art lessons; taking music lessons, etc.), and self-improvement (reading; doing homework; doing a chore, etc.). The activities are then divided into two domains: formal (structured activities with a coach, as doing gymnastics, learning to dance, etc.) and informal activities (have little planning and are often initiated by oneself (e.g., reading, hanging out). The child reports how much he/she (1) really likes to do the activity; (2) sort of likes to do; and (3) does not like to do the activity. Although the PAC was designed for children aged 6–21 years, it has been in use with younger ages [25,26].

The Short Sensory Profile (SSP) [27,28]—This parent-report contains seven sections that evaluate behaviors associated with abnormal responses to sensory stimuli in daily life and in all sensory modalities (for example: “avoids certain tastes or food smells that are typically part of children’s diets”; “avoids going barefoot, especially in sand or grass”). Parents mark the frequency of the behavior in daily life, on a Likert scale ranging from 1 (always) to 5 (never). The total scale is 38 to 190, with higher scores (155–190) reflecting normal performance. In the typical population, 2% of the children are found in the “Definite Difference” range (two standard deviations below the mean); 14% in the “Probable Difference” score (between one-two standard deviation below the mean) and 84% are found in the normal range.

### 2.3. Procedure

After receiving approval from the ethics committee of [BLINDED], the parents of all children filled out a consent form approving their participation with their child. A meeting was arranged with the parents of children in their home or in the clinic. Parents completed the SSP while their children completed the PAC.

### 2.4. Data Analysis

Data was analyzed using SPSS-27. Chi square (followed y z-test and Bonferroni test) and One Way ANOVA (with Bonferroni post hoc test) examined differences in socio-demographic parameters between groups. Chi square also examined differences between the groups regarding the percentage of children found in each of the SSP performance range. Two way MANCOVA (for subscales) and two way ANCOVA (for total scores) examined the differences between groups and genders in SSP and PAC, while age was held as a covariate. The correlations between SSP and PAC scores were examined by Pearson test. The significance level was set on *p* ≤ 0.05.

## 3. Results

### 3.1. Hypothesis 1: Differences Between Groups in Preference to Participate in Activities

#### 3.1.1. Differences Between Healthy Controls and the Three Atopic Groups

Healthy controls had significant higher preference to participate in activities then the whole group of atopic diseases (Table 2). When referring to each atopic group, according to Bonferroni post hoc, the controls showed significantly higher preference to participate in activities (PAC total score) than children with asthma (*p* ≤ 0.0001) and children with AR (*p* = 0.02), but not from children with AD.

#### 3.1.2. Differences in PAC Between Healthy Controls and Each Atopic Group

When referring to PAC scales, representing various types of daily activities, healthy controls had greater preferences to participate in activities than children with Asthma. This was found in recreational (*p* = 0.01); active physical (*p* = 0.002); skill-based (*p* < 0.0001); self-improvement (*p* = 0.03); formal (*p* < 0.0001) and informal activities (*p* = 0.001). Healthy controls had also greater preferences to participate in activities than children with AR as found in social (*p* = 0.03); skill based (*p* = 0.02); self-improvement (*p* = 0.03) and informal activities (*p* = 0.005).

Children with AD showed a significantly lower preference to participate in active physical, skilled-based and formal activities than healthy controls (as reported in Engel-Yeger et al.) [8].

### 3.2. Hypothesis 2: Differences Between the Three Atopic Groups in Their Preference to Participate in Activities

Children with AD had a higher preference to participate in activities as compared to children with Asthma—in recreational activities (*p* = 0.04) and in informal activities (*p* = 0.016) (see Table 3).

#### Gender Effect

When comparing all boys and all girls, from both—healthy and atopic groups, girls had significantly higher preference to participate in skill-based activities than the boys (1.64 ± 0.43; 1.98 ± 0.49, respectively) (F_1,205_ = 30.17, *p* < 0.001). Similar trend was found in self-improvement activities (1.83 ± 0.41; 1.95 ± 0.47, respectively) (F_1,205_ = 4.59, *p* = 0.03); informal activities (1.52 ± 0.28; 1.61 ± 0.27, respectively) (F_1,205_ = 5.88, *p* = 0.016) and in the total PAC score (1.61 ± 0.27; 1.68 ± 0.31, respectively) (F_1,205_ = 3.91, *p* = 0.048). However, the boys showed significant higher preference to participate in active physical activities than the girls (1.58 ± 0.44; 1.78 ± 0.46, respectively) (F_1,205_ = 9.38, *p* = 0.002).

When comparing healthy children to the whole atopic group, boys with atopic diseases had the lowest preference (2.19 ± 0.45) as compared to healthy boys (1.81 ± 0.44), healthy girls (1.62 ± 0.39) and girls with atopic diseases (1.67 ± 0.48). Similar trend was found in formal activities (F_1,205_ = 4.46, *p* = 0.036), where boys with atopic diseases had the lowest preference (2.07 ± 0.39) as compared to healthy boys (1.74 ± 0.41), healthy girls (1.79 ± 0.37) and girls with atopic diseases (1.89 ± 0.44).

When referring to healthy children and the three atopic groups, girls with AD showed the greatest preference to participate in skill-based and in formal activities than boys and girls from all other atopic groups, and from the controls. Boys with Asthma showed the lowest preference to participate in these activity types than all boys and girls in the other atopic groups as well as from the controls (see Table 4).

### 3.3. Hypothesis 3: The Correlations Between SMD and the Preference to Participate in Daily Activities Among Children with Atopic Diseases SMD

First, we examined the prevalence of SMD and found a significantly higher percentage of SMD among children with atopic diseases as compared to healthy controls, in all SSP scales. The highest prevalence of children found in the abnormal “*definite difference*” range was in children with AD (in the tactile, visual/auditory sensitivity; auditory filtering and seeking scales), as compared to two scales among the asthmatic children (seeking and auditory filtering) and two scales among children with AR (taste/smell sensitivity; low energy). Table 5 depicts the percentage of children found in each SSP range.

Based on Bonferroni post hoc test, no significant differences were in SSP scores between the three atopic groups. However, each atopic group had significantly worse scores as compared to the healthy controls (Table 6).

In each atopic group, the highest number of correlations was found in the AD and the asthma groups. Among children with AD, preference to participate in recreational activities positively correlated with lower auditory filtering abilities (r = 0.42, *p* = 0.03) and with greater visual/auditory sensitivity (r = 0.44, *p* = 0.02). Among children with asthma, higher sensitivity to vestibular stimuli significantly correlated with elevated preference to participate in activities according to PAC total score (r = 0.39, *p* = 0.02) and PAC scales: skill-based (r = 0.36, *p* = 0.04) and informal activities (r = 0.37, *p* = 0.03). No significant correlations were found between SSP and PAC scores among children with AR.

**Figure 1:**
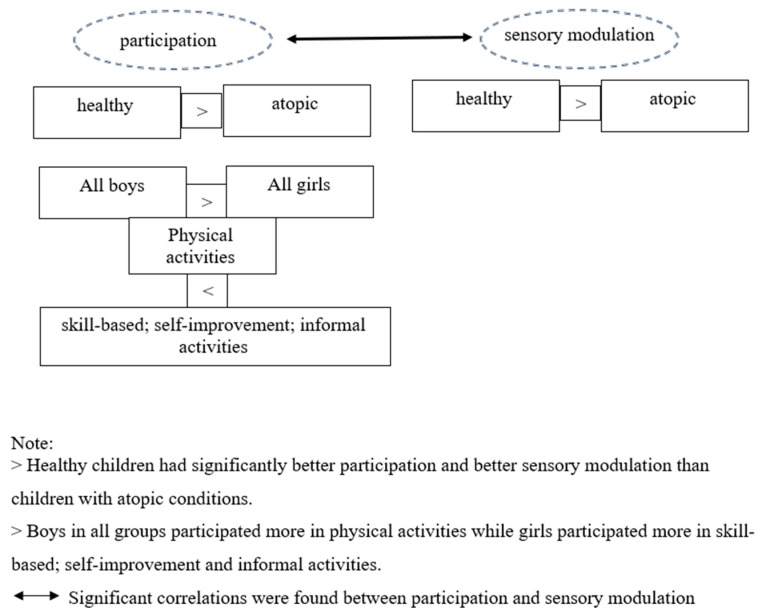
Summary of main results.

Figure 1 summarizes the main results.

## 4. Discussion

This present study aimed to compare the preference to participate in daily activities between children with AD, asthma and AR and healthy controls. This study illuminated unique participation profiles of each atopic group. Based on the high prevalence of SMD in children with atopic diseases, the study also presented the prevalence of SMD in the three atopic groups and examined the relationships between SMD and the preference to participate in daily activities.

As hypothesized in hypothesis 1, preference to participate in activities was significantly lower among children with atopic diseases as compared to healthy controls. This was manifested mainly in physical and social activities [13,29]. Nonetheless, gaps between groups also exist in other activity types—recreational, skill-based; self-improvement; formal and informal activities. Studies show that the chronicity and complexity of atopic diseases might reduce children’s physical and emotional status, impair self-esteem and social interactions [30,31,32]. These outcomes may cause children to limit their participation in leisure activities with peers, Therefore, a thorough evaluation of the way the disease impacts children’s preference to participation in daily activities is essential.

As hypothesized in hypothesis 2, within the atopic groups, similar preference to participate in activities was found in most activity types. Yet, children with AD had a higher preference to participate in recreational and in informal activities (activities that not necessarily involve a tutor, such as: playing games, playing non-team sport; listening to music) as compared to children with Asthma. Recreational activities are mainly quiet activities (doing crafts, drawing, collecting things, playing on the computer, doing puzzles). The physical appearance of children with AD may cause stigmatization, embarrassment and social isolation or avoidance from social interactions, because peers might express disgust and fear of playing with them [33]. It may be suggested that in these activities, children with AD control better their inflamed skin, as compared to physical activities in which the child may sweat, or water sports activities, which may irritate the skin [29,30]. Nguyen et al. [33] noted that AD may cause negative behavioral effects on children and reduce their opportunity to develop proper coping. Recreational activities, that can be performed alone, may reflect the preference to minimize interaction with peers. In asthma and AR body appearance may not necessarily be affected and therefore may not deteriorate child’s self-esteem and confidence when interacting with peers, as in children with AD.

This study adds knowledge about the interaction between preference to participate in daily activities and gender. In general, when referring to all children, girls had significantly higher preference to participate in skill-based, self-improvement and informal activities than the boys. Boys showed a significantly higher preference to participate in active physical activities than the girls. This supports previous reports on healthy children and on children with disabilities, showing that girls prefer to participate more in social and in skill-based activities, while boys prefer more active physical activities [2,15].

Interestingly, when dividing the participants into healthy and atopic groups, boys with atopic diseases had the lowest preference to participate in activities. However, this was found only in regard to skill-based (as playing a musical instrument; taking art lessons) and formal activities. This result may strengthen the reports about boys that prefer physical activities rather than skill-based activities. When referring to each atopic group, girls with AD showed the greatest preference to participate in skill-based and in formal activities than boys and girls from all other atopic groups, and the controls. The irritated skin in AD and the possible embarrassing appearance may be more emphasized in girls than in boys with AD. Indeed, AD in girls was reported to have more prominent effects on girls’ self-esteem and self-confidence as compared to the boys [33]. Thus, girls with AD may prefer to participate in skill-based activities that can be performed alone and not necessarily with peers. However, based on the small sample, further studies should examine gender effect on participation.

When referring to hypothesis 3, regarding the relationships between SMD of children with atopic diseases, SMD prevalence was similar in the three atopic groups. About 30% of the children in each group were found in the “definite difference” range (as compared to about 6% of the healthy controls). Yet, the different prevalence of children found in the abnormal sensory performance ranges on each modality/SSP scale (as in movement sensitivity; low energy) suggests that each atopic group is more vulnerable to SMD in specific modalities and in a different severity level than the other two groups.

In each atopic group, SMD correlated with reduced activity preference: children with asthma with higher sensitivity to vestibular stimuli preferred skill-based (swimming; dancing) and informal activities. As mentioned above, skill-based activities include quiet activities that can be performed alone. This emphasizes the danger in isolation, loneliness and in deteriorating a child’s emotional and functional wellbeing [34,35]. Since comorbidities as SMD may further deteriorate child’s participation, SMD should be screened SMD and if found, examine implications on child’s daily activities [15]. Receiving information from both parents (such as via the SSP) and children (via PAC) may provide a deeper perspective about the disease/comorbidities implications on obstacles in child’s daily life. Intervention should aim to enhance opportunities for children’s participation with peers in daily life settings, such as home, school and community. This can be achieved by increasing teachers’ and parents’ awareness of the challenges children face, thereby creating more opportunities for participation both in school and in the community.

Practically, physicians should suggest parents and children to explore ways to enhance participation across various domains. This includes activities that provide sensory stimulation and promote peer interactions, such as physical activities in the community (e.g., swimming, martial arts, team sports), in school (e.g., sports during recess); self-improvement and skill-based activities at home (like art projects), in school (creating social platforms for peer activities like gardening in the schoolyard). By doing so, we can improve children’s coping skills, physical and emotional well-being, and overall quality of life.

## 5. Conclusions

Atopic diseases might restrict children’s participation and be related to comorbidities as sensory modulation difficulties. Health providers should refer to the possible negative impacts of atopic diseases on children’s participation with a vast perspective: examine which participation patterns are restricted, how they relate to physical and emotional status [31,32,33,34], to SMD, to clinical manifestations atopic diseases (as breathing in asthma and AR; irritated skin in AD), comorbidities as nasal symptoms (22.3% vs. 37.8%) and sleep disturbance in children with AD, asthma and AR [32,34]. Participation should be evaluated and considered as a main outcome measure of intervention efficiency and the balance of the disease. By understanding the implications of atopic diseases on children’s participation in daily life, we may better focus intervention on children’s experiences and involvement in real life settings, including with peers. By that, we can minimize negative functional and emotional effects, enhance optimal development and child’s wellbeing.

## 6. Research Limitations

This study includes a relatively small number of participants in the atopic groups (even smaller number than recommended by G-Power) with unequal number of participants in the three atopic groups; unequal distribution of socio-demographic parameters, within and between groups, as gender; the study referred to a specific age group. This study did not differentiate patients by disease severity although disease severity could impact participation. Further studies should examine the relation between participation and sensory modulation difficulties in additional age groups, with respect to disease severity, and in larger sample sizes to generalize the results.

## Figures and Tables

**Table 1:** Participants’ demographic information.

		AD (*n* = 37)	Asthma (*n* = 37)	AR (*n* = 31)	Controls (*n* = 148)		*p*
Age range		4–11	4–11	5–11	4–10.6		
Mean age ± SD		6.71 ± 1.91	8.37 ± 1.81	9.53 ± 1.39	7.59 ± 1.55	F_3,241_ = 18.09	<0.001
Gender—n (%)	Boys	14 (37.8%)	23 (62.2%)	22 (71%)	78 (52.7%)	Χ^2^_3_ = 8.58	0.035
	Girls	23 (62.2%)	14 (37.8%)	9 (29%)	70 (47/3%)		
Father’s education	High school	10 (27%)	6 (16.2%)	2 (6.5%)	0	Χ^2^_3_ = 30.62	<0.001
	Professional	3 (8.1%)	5 (13.5%)	7 (22.6%)	28 (18.9%)		
	Academy	17 (45.9%)	24 (64.9%)	17 (54.8%)	101 (68.2%)		
	Missing	7 (18.8%)	2 (5.4%)	5 (16.1%)	19 (12.9%)		
Mother’s education	High school	9 (24.3%)	1 (2.7%)	3 (9.7%)	0	Χ^2^_3_ = 21.37	0.002
	Professional	3 (8.2%)	1 (2.7%)	5 (16.1%)	29 (19.6)		
	Academy	19 (51.3)	25 (67.6%)	19 (61.3%)	82 (55.4)		
	Missing	6 (16.2%)	10 (27%)	4 (12.9%)	37 (25%)		

Note: AD, Atopic Dermatitis; AR, Allergic Rhinitis; SD, standard deviation.

**Table 2:** Differences in preference to participate in activities between healthy controls and the whole group of atopic diseases.

	All Atopic Diseases		Healthy Controls				
	Mean	SD	Mean	SD	F_1,205_	*p*	Eta^2^
Recreational activities	1.56	0.29	1.47	0.28	5.88	0.016	0.03
Active physical activities	1.81	0.45	1.56	0.44	13.89	<0.001	0.07
Social Activities	1.48	0.36	1.35	0.28	8.58	0.004	0.03
Skill Based activities	1.98	0.53	1.72	0.43	16.83	<0.001	0.06
Self-improvement activities	1.97	0.46	1.84	0.43	6.37	0.012	0.03
Formal activities	2.01	0.42	1.76	0.39	17.43	<0.001	0.07
Informal activities	1.63	0.27	1.52	0.25	9.72	0.002	0.05
Total activities	1.73	0.29	1.58	0.27	14.19	<0.001	0.06

Note: Higher scores represent lower preference; SD = standard deviation.

**Table 3:** Comparing PAC scores between all groups.

	Asthma		AD		AR		Controls			
	Mean	SD	Mean	SD	Mean	SD	Mean	SD	F_3,207_	Eta^2^
Recreational activities	1.65	0.25	1.45	0.32	1.56	0.29	1.47	0.28	4.43 **	0.06
Active physical activities	1.84	0.37	1.75	0.51	1.77	0.45	1.56	0.44	5.21 **	0.07
Social Activities	1.51	0.34	1.41	0.43	1.55	0.32	1.35	0.28	3.64 **	0.06
Skill Based activities	2.06	0.54	1.91	0.54	1.96	0.51	1.72	0.43	4.99 **	0.08
Self-improvement activities	2.04	0.47	1.78	0.43	2.09	0.41	1.84	0.43	4.75 **	0.06
Formal activities	2.09	0.39	1.93	0.51	1.95	0.36	1.76	0.39	5.93 ***	0.09
Informal activities	1.71	0.24	1.51	0.31	1.68	0.24	1.52	0.25	6.92 ***	0.1
Total activities	1.81	0.27	1.61	0.33	1.75	0.26	1.58	0.27	6.23 ***	0.1

Note: Higher scores represent lower preference. PAC, Preference for Activities of Children; SD, standard deviation; AD, Atopic Dermatitis; AR, Allergic Rhinitis; ** *p* ≤ 0.01; *** *p* ≤ 0.001.

**Table 4:** Comparing PAC skill-based and formal activity scores between genders.

		Asthma		AD		AR		Controls			
		Mean	SD	Mean	SD	Mean	SD	Mean	SD	F_1,201_	Eta^2^
Skill Based activities	Boys	*2.33*	0.38	2.27	0.41	2.01	0.51	1.81	0.49	4.67 **	0.13
	Girls	1.62	0.48	1.61	0.45	*1.84*	0.56	1.62	0.39		
Formal activities	Boys	*2.24*	0.34	2.06	0.49	1.91	0.34	1.74	0.41	3.87 **	0.006
	Girls	1.85	0.38	1.84	0.51	*2.05*	0.41	1.79	0.37		

Note: PAC, Preference for Activities of Children; SD, standard deviation; AD, Atopic Dermatitis; AR, Allergic Rhinitis; ** *p* ≤ 0.01; Italic = Less preferable.

**Table 5:** The percentage of children in each SSP range.

	All Atopic Groups			Controls			Chi^2^	*p*
SSP Sections	=	+	++	=	+	++		
Tactile Sensitivity	53.1	18.4	28.5	73.9	14.2	11.9	12.65	0.002
Taste/Smell Sensitivity	74.6	17.2	8.2	74.6	17.2	8.2	19.49	<0.001
Movement Sensitivity	66.3	13.3	20.4	61.5	17.9	4.5	14.57	<0.001
Under Responsive/Seek	50.5	20.6	28.9	74.6	14.9	10.4	16.23	<0.001
Auditory Filtering	60.8	22.7	16.5	81.3	14.2	4.5	14.08	0.001
Low Energy/Weak	59.4	17.7	22.9	79.9	11.2	9	12.37	0.002
Visual/Auditory Sensitivity	79.2	10.4	10.4	90.3	7.5	2.2	7.98	0.018
Total	55.1	12.2	32.7	80.6	12.7	6.7	26.82	<0.001

Note: SSP, Short Sensory Profile; = Typical performance; + Probable difference; ++ Definite difference.

**Table 6:** Comparing SSP scores between healthy controls and the three atopic groups.

	AD		Asthma		AR		Controls		F_3,226_	*p*	Eta^2^
SSP Sections	Mean	SD	Mean	SD	Mean	SD	Mean	SD			
Tactile Sensitivity	27.13	6.99	29.17	4.81	29.69	3.82	31.06	3.92	7.62	<0.001	0.09
Taste/Smell Sensitivity	14.19	5.54	14.28	4.54	13.88	4.58	16.55	3.42	6.28	<0.001	0.08
Movement Sensitivity	13.41	2.45	12.25	3.35	12.19	3.01	13.71	1.78	5.12	0.002	0.06
Under Responsive/Seek	26.16	6.64	27.67	4.96	26.69	5.19	29.68	4.29	7.04	<0.001	0.09
Auditory Filtering	23.21	5.17	24.05	4.24	23.57	4.64	25.61	3.49	4.67	0.003	0.06
Low Energy/Weak	26.43	4.27	26.03	4.61	24.26	4.47	27.91	2.92	8.84	<0.001	0.11
Visual/Auditory Sensitivity	19.75	5.46	21.72	4.62	21.69	3.13	22.72	2.66	6.67	<0.001	0.08
Total	150.29	28.91	151.01	26.77	152.01	18.59	167.25	15.71	11.87	<0.001	0.14

Note: Lower scores represent worse sensory modulation. SD, standard deviation; AD, Atopic Dermatitis; AR, Allergic Rhinitis.

## Data Availability

Data is unavailable due to privacy and ethical restrictions.
